# Adrenal myelolipoma with severe hemorrhage: a case report

**DOI:** 10.1093/jscr/rjag807

**Published:** 2026-09-22

**Authors:** Taylor Messick-Ngo, Jasmine Kazemi Gwizdala, Manika Paul, Ahmed Mahmoud, Gustavo Lara

**Affiliations:** Department of General Surgery, Riverside Community Hospital, 4445 Magnolia Ave, Riverside, CA 92501, United States; Riverside School of Medicine, University of California, 92521 Botanic Gardens Dr, Riverside, CA 92507, United States; Department of General Surgery, Riverside Community Hospital, 4445 Magnolia Ave, Riverside, CA 92501, United States; Department of General Surgery, Riverside Community Hospital, 4445 Magnolia Ave, Riverside, CA 92501, United States; Department of General Surgery, Riverside Community Hospital, 4445 Magnolia Ave, Riverside, CA 92501, United States

**Keywords:** adrenal myelolipoma, retroperitoneal hemorrhage, adrenal incidentaloma, adrenalectomy, hemorrhagic adrenal mass, retroperitoneal tumor

## Abstract

Adrenal masses are identified with abdominal imaging, most of them are benign and nonfunctioning. They are typically managed conservatively; however, large lesions may present with complications requiring urgent intervention. We present a case of a 65-year-old male with right flank pain found to have a rapidly enlarging hemorrhagic right adrenal mass measuring 15.4 × 13.1 × 17.9 cm with mixed fat and soft tissue components. Hormonal evaluation was negative for a functional adrenal tumor, though malignancy could not be excluded due to ongoing hemorrhage and lesion size. The patient required blood transfusion and open adrenalectomy with en bloc right colectomy. The mass was densely adherent to surrounding structures with significant clot burden. Final pathology confirmed an adrenal myelolipoma composed of mature adipose tissue and tri-lineage hematopoietic elements with extensive intra-tumoral hemorrhage and necrosis. This case highlights the importance of prompt surgical management of large hemorrhagic adrenal masses.

## Introduction

Adrenal tumors are identified incidentally with the widespread use of abdominal imaging, particularly in older populations. Approximately 10% of patients over 60 years of age are found to have adrenal lesions, the majority of which are benign and nonfunctioning [1].

Adrenal myelolipomas are rare benign mesenchymal tumors composed of mature adipose tissue and tri-lineage hematopoietic tissue, accounting for ~3%–5% of primary adrenal neoplasms [2]. Lesions greater than 4 cm are most commonly nonfunctioning adenomas or benign functioning tumors; however, increasing size is associated with a higher risk of hemorrhage and malignancy. Although most adrenal myelolipomas are asymptomatic and discovered incidentally, larger lesions (>6–7 cm) carry an increased risk of spontaneous hemorrhage, rupture, and compressive symptoms, making surgical intervention appropriate in selected patients.

We present a case of a large adrenal myelolipoma complicated by acute hemorrhage requiring urgent surgical intervention.

## Case presentation

A 65-year-old male presented to the emergency department with right flank pain and headaches for 1 week. His medical history was significant for deep vein thrombosis treated with clopidogrel and an inferior vena cava filter. He had no history of malignancy.

**Figure 1:**
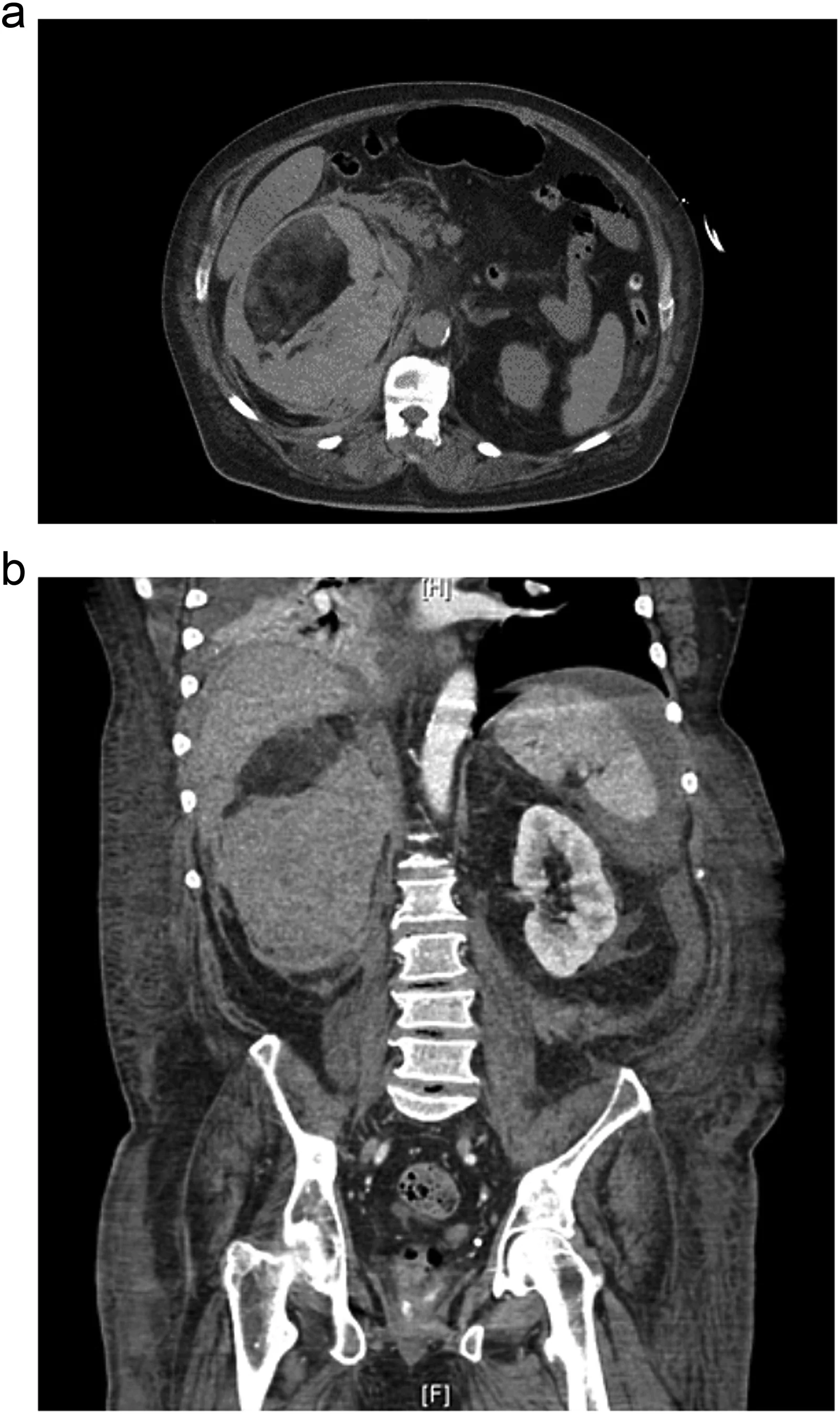
Axial (a) and coronal (b) contrast-enhanced CT images demonstrating a large heterogeneous right adrenal mass with mixed fat and soft tissue components and surrounding hyperdense areas consistent with acute hemorrhage.

Initial imaging revealed a 5.8 cm right adrenal mass with associated hemorrhage, and the patient was transferred to a facility with interventional radiology capabilities. Repeat contrast-enhanced computed tomography (CT) demonstrated a 15.4 × 13.1 × 17.9 cm heterogeneous right adrenal mass containing fat and soft tissue components (Fig. 1a and b). Angiography demonstrated no active bleeding, and embolization was not performed.

The patient required resuscitation with four units of packed red blood cells and one unit of platelets prior to surgery. Comprehensive hormonal evaluation, including adrenal, thyroid, and catecholamine studies, was normal.

Following discussion with radiology, the mass was suspected to represent a myelolipoma; however, a definitive diagnosis could not be established due to distortion from hemorrhage. Given its large size and malignancy risk, the decision was made to proceed with open adrenalectomy with possible right colon resection.

**Figure 2:**
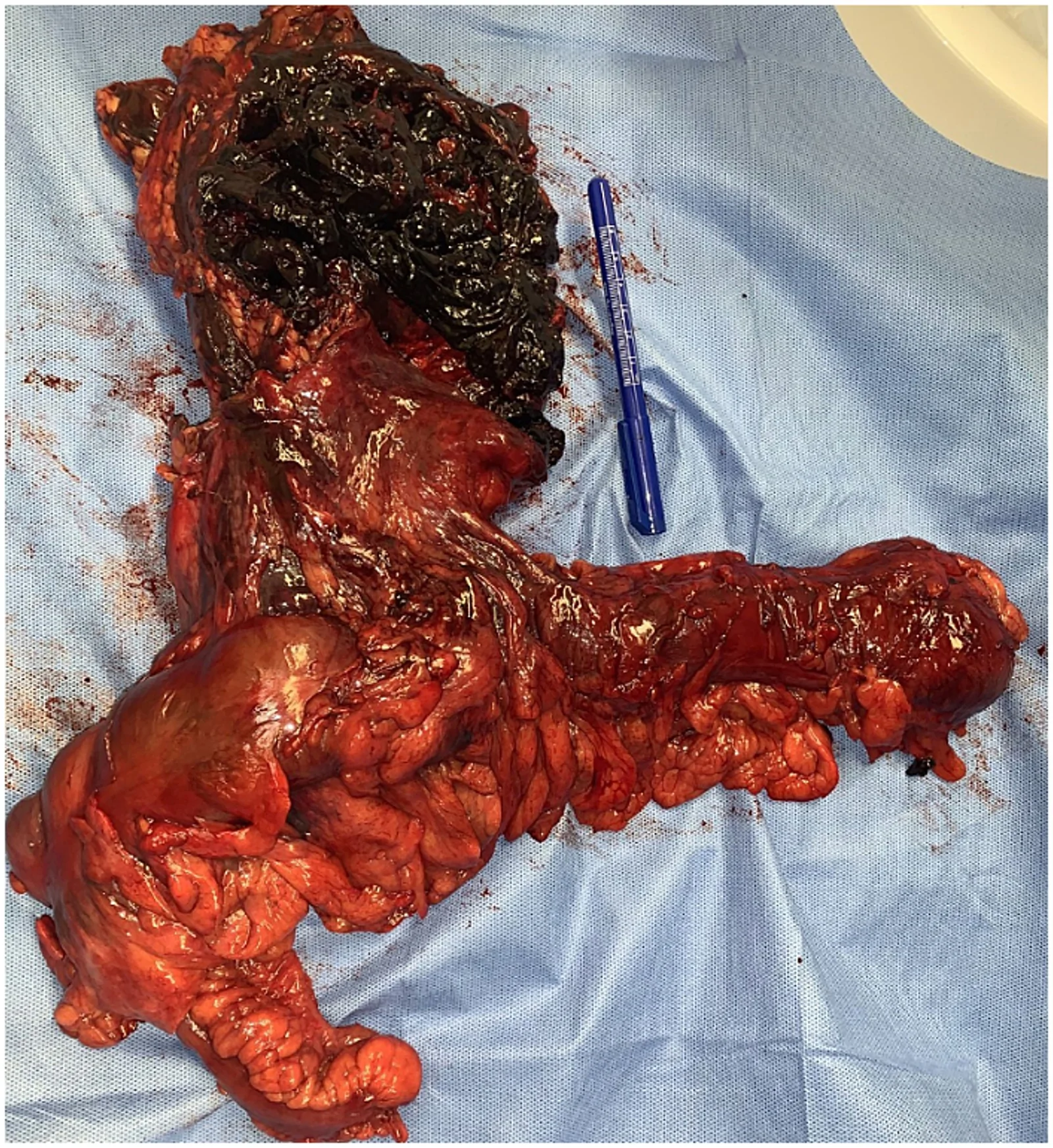
Gross specimen demonstrating en bloc resection of a large right adrenal myelolipoma with adherent right colon, showing extensive hemorrhagic and necrotic components.

A midline laparotomy was performed. Old blood clots were encountered on entry, consistent with prior intra-peritoneal hemorrhage. The right colon and mesocolon were densely adherent to the mass, necessitating an en bloc right colectomy with primary anastomosis using a gastrointestinal anastomosis stapler (Fig. 2).

The mass was mobilized from the diaphragm, kidney, inferior vena cava, and surrounding structures. Multiple clots confirmed prior hemorrhage. The right adrenal vein was not visualized because of distortion. Vascular control was achieved using LigaSure, and a retroperitoneal drain was placed.

The postoperative course was uncomplicated, and the patient was discharged on postoperative Day 6.

**Figure 3:**
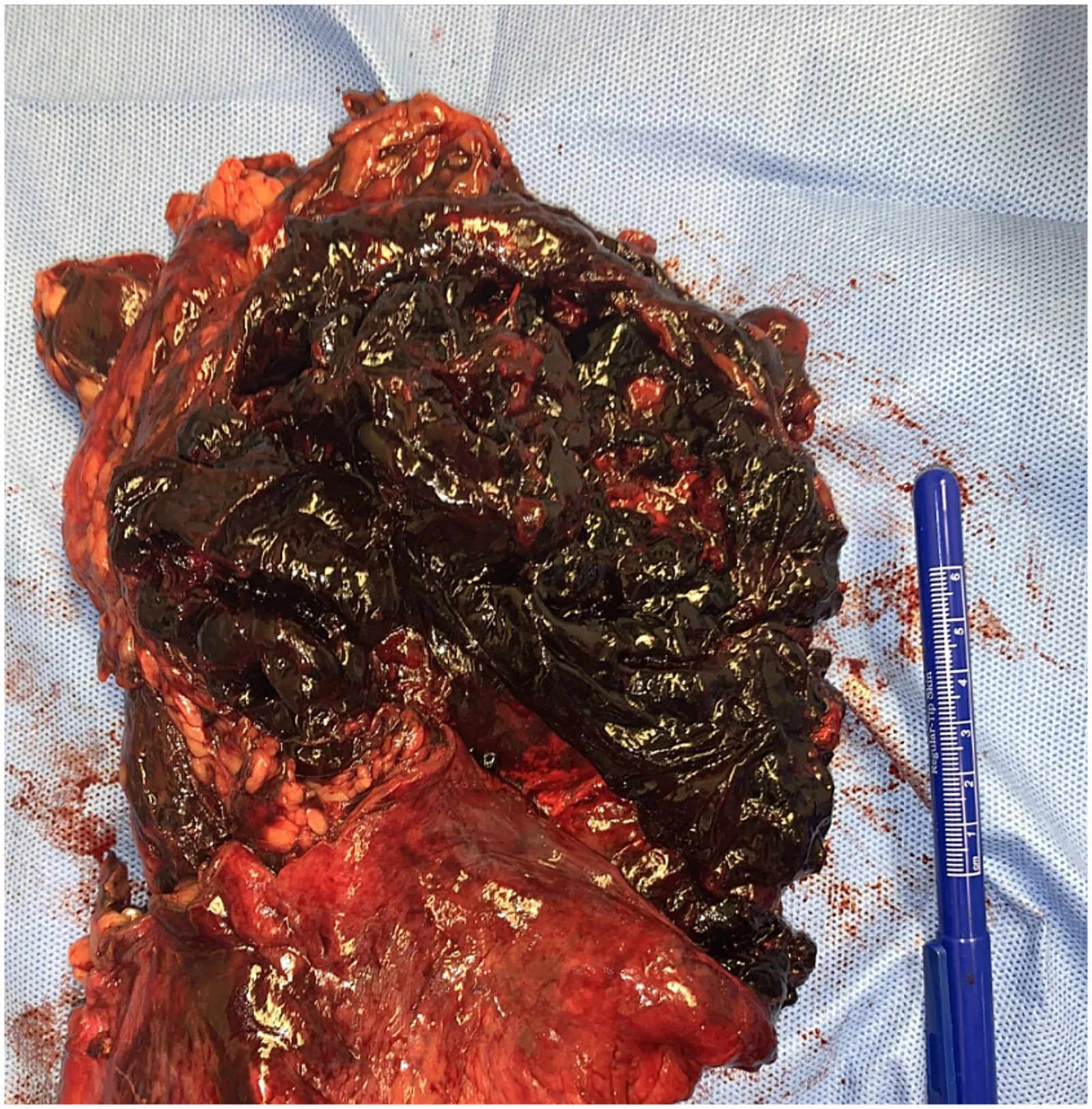
Gross specimen of right adrenal myelolipoma demonstrating extensive intra-tumoral hematoma with areas of hemorrhage and necrosis.

**Figure 4:**
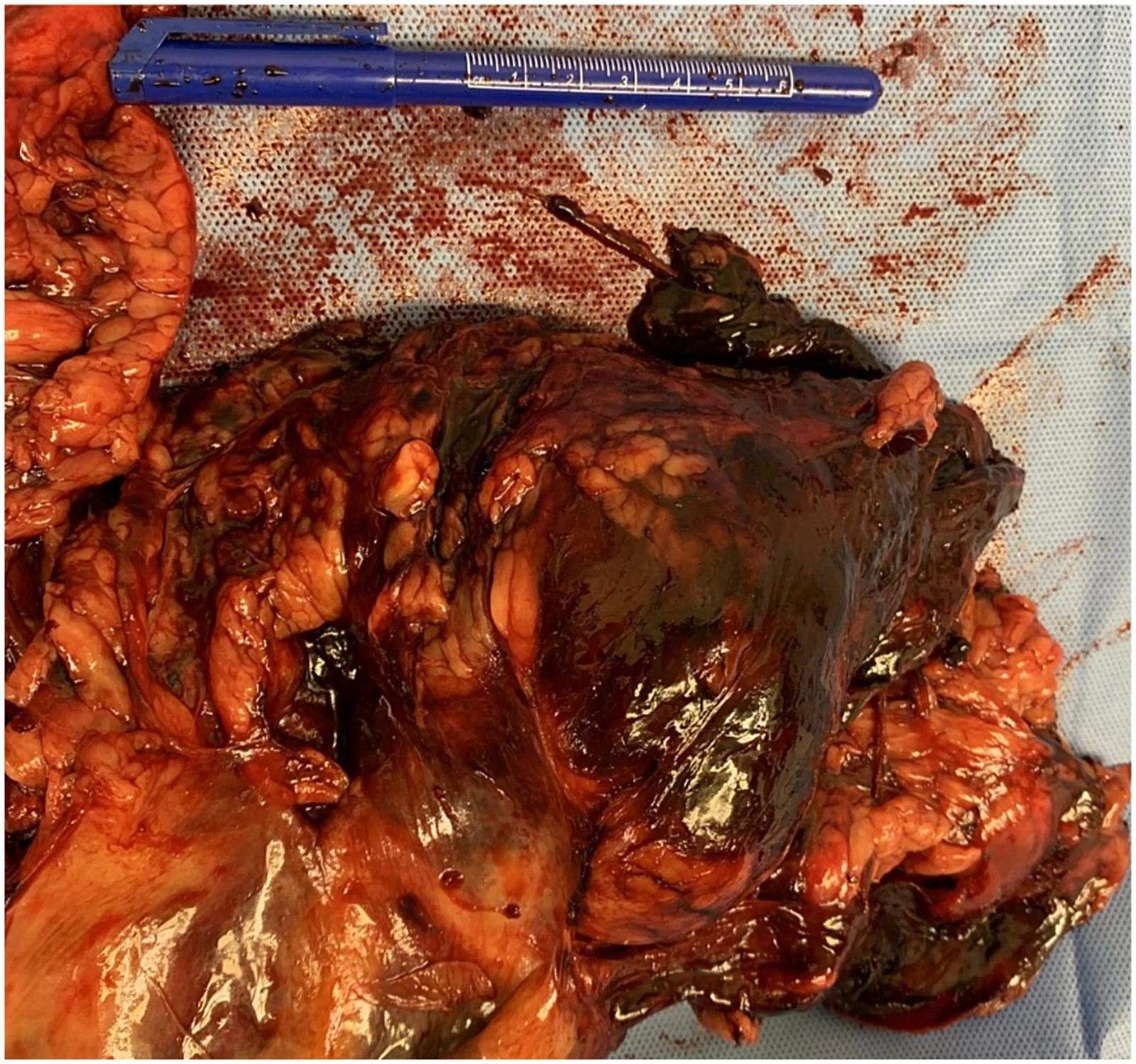
Gross specimen of right adrenal myelolipoma following evacuation of intra-tumoral hematoma, demonstrating tumor architecture.

Final histopathologic examination demonstrated a benign adrenal myelolipoma composed of mature adipose tissue admixed with tri-lineage hematopoietic elements, with extensive intra-tumoral hemorrhage and necrosis (Figs 3 and 4).

## Discussion

Adrenal tumors are increasingly identified with widespread abdominal imaging, the majority of which are benign and nonfunctioning [3]. This case is notable for spontaneous hemorrhage. In this case, the tumor measured 17.9 cm, and percutaneous biopsy was not pursued due to increased risk associated with lesions greater than 6 cm [4].

Adrenal myelolipomas are rare benign mesenchymal neoplasms composed of mature adipose tissue and tri-lineage hematopoietic elements, accounting for ~3%–5% of all primary adrenal neoplasms [2]. They are typically nonfunctioning and discovered incidentally during abdominal imaging, with most patients remaining asymptomatic throughout their lifetime [2, 5]. Although the exact pathogenesis remains uncertain, the leading hypothesis suggests metaplastic transformation of adrenal cortical cells in response to chronic stress, inflammation, infection, or necrosis [2].

Characteristic CT findings include macroscopic fat interspersed with soft-tissue attenuation [6]. However, extensive hemorrhage, necrosis, or rapid interval enlargement may obscure these characteristic imaging features, making differentiation from adrenocortical carcinoma, retroperitoneal liposarcoma, or other adrenal malignancies more challenging [6].

Management of adrenal myelolipomas is guided primarily by symptoms, tumor size, interval growth, and diagnostic uncertainty rather than malignant potential, as these lesions do not undergo malignant transformation. Small asymptomatic lesions may be managed conservatively with surveillance imaging, whereas surgical resection is recommended for symptomatic tumors, enlarging lesions, indeterminate imaging findings, or tumors larger than ~6–7 cm because of the increased risk of spontaneous hemorrhage and rupture [5, 7].

Although adrenal myelolipomas are typically nonfunctioning, endocrine evaluation remains important to exclude coexisting functional adrenal neoplasms before surgery [7]. In this case, hormonal evaluation was unremarkable, and imaging findings were consistent with myelolipoma.

Our patient’s clinical course illustrates an uncommon but potentially life-threatening presentation of adrenal myelolipoma. The lesion rapidly enlarged to 17.9 cm, developed extensive spontaneous hemorrhage requiring blood transfusion, and demonstrated dense adherence to adjacent structures, ultimately necessitating open adrenalectomy with en bloc right colectomy. Histopathology confirmed mature adipose tissue admixed with tri-lineage hematopoietic elements and extensive hemorrhage and necrosis.

Although adrenal myelolipomas have an excellent prognosis after complete resection, giant hemorrhagic lesions may present with life-threatening complications requiring urgent surgery. Early endocrine evaluation and timely surgical intervention are essential in giant hemorrhagic myelolipomas.
